# Linking resting-state network fluctuations with systems of coherent synaptic density: A multimodal fMRI and ^11^C-UCB-J PET study

**DOI:** 10.3389/fnhum.2023.1124254

**Published:** 2023-02-23

**Authors:** Xiaotian T. Fang, Tommaso Volpi, Sophie E. Holmes, Irina Esterlis, Richard E. Carson, Patrick D. Worhunsky

**Affiliations:** ^1^Department of Radiology & Biomedical Imaging, Yale School of Medicine, New Haven, CT, United States; ^2^Department of Psychiatry, Yale School of Medicine, New Haven, CT, United States; ^3^Department of Psychology, Yale University, New Haven, CT, United States

**Keywords:** PET, resting-state network (RSN), fMRI, multimodal neuroimaging, ^11^C-UCB-J, SV2A, ICA

## Abstract

**Introduction:** Resting-state network (RSN) connectivity is a widely used measure of the brain’s functional organization in health and disease; however, little is known regarding the underlying neurophysiology of RSNs. The aim of the current study was to investigate associations between RSN connectivity and synaptic density assessed using the synaptic vesicle glycoprotein 2A radioligand ^11^C-UCB-J PET.

**Methods:** Independent component analyses (ICA) were performed on resting-state fMRI and PET data from 34 healthy adult participants (16F, mean age: 46 ± 15 years) to identify *a priori* RSNs of interest (default-mode, right frontoparietal executive-control, salience, and sensorimotor networks) and select sources of ^11^C-UCB-J variability (medial prefrontal, striatal, and medial parietal). Pairwise correlations were performed to examine potential intermodal associations between the fractional amplitude of low-frequency fluctuations (fALFF) of RSNs and subject loadings of ^11^C-UCB-J source networks both locally and along known anatomical and functional pathways.

**Results:** Greater medial prefrontal synaptic density was associated with greater fALFF of the anterior default-mode, posterior default-mode, and executive-control networks. Greater striatal synaptic density was associated with greater fALFF of the anterior default-mode and salience networks. *Post-hoc* mediation analyses exploring relationships between aging, synaptic density, and RSN activity revealed a significant indirect effect of greater age on fALFF of the anterior default-mode network mediated by the medial prefrontal ^11^C-UCB-J source.

**Discussion:** RSN functional connectivity may be linked to synaptic architecture through multiple local and circuit-based associations. Findings regarding healthy aging, lower prefrontal synaptic density, and lower default-mode activity provide initial evidence of a neurophysiological link between RSN activity and local synaptic density, which may have relevance in neurodegenerative and psychiatric disorders.

## 1 Introduction

Resting-state functional MRI (fMRI) connectivity has become a widely used tool for understanding the brain’s functional organization. Since the initial observations of task-independent, synchronous low-frequency fluctuations in blood oxygen level-dependent (BOLD) fMRI signal (Raichle et al., 2001), a number of canonical resting-state networks (RSNs) that also display functional specificity during cognitive tasks have been identified (Smith et al., 2009; Laird et al., 2011; Barkhof et al., 2014). While this has significantly advanced the understanding of the functional architecture of the brain, less is known about the underlying neurophysiology of these RSNs. Understanding the cell-level factors associated with RSN functioning would provide valuable insight into their neurobiological bases, which could in turn prove vital in understanding certain disease states.

Alterations in RSN functioning have been reported in relation to biological processes and diseases with established neurophysiological trajectories. RSNs including the default-mode, executive-control, sensorimotor, and salience networks, display reductions in functional activity associated with healthy aging and neurodegenerative diseases (Wu et al., 2011; Li et al., 2012; Pasquini et al., 2015; Varangis et al., 2019; Wolters et al., 2019). Similarly, altered RSN activity has been reported in psychiatric disorders that are also characterized by macrostructural brain abnormalities, including schizophrenia and substance-use disorders (Ma et al., 2010; Li et al., 2019; Zhou et al., 2019). However, the manner and degree to which neuronal factors such as synaptic density contribute to RSN functioning remain largely unknown.

The magnitude and temporal characteristics of BOLD signals have been linked in part to the relative composition of gray matter, white matter, and vasculature within a given voxel at the macrostructural level (Provencher et al., 2018). In addition to the influences of local tissue properties on BOLD signal variability, multiple pathways involving cortical and subcortical regions are likely to play critical roles in modulating the neural activity of RSNs (Macpherson et al., 2014). Indeed, the anatomical bases for cortico-subcortical circuits have been identified using diffusion tensor imaging, including fiber tracts between the striatum and cortical regions associated with the default-mode and executive functioning networks (Lehéricy et al., 2004a, b; Leh et al., 2007; Alves et al., 2019). Furthermore, simultaneous PET/MRI studies have demonstrated that stimulus-evoked molecular activity at synapses does not necessarily co-localize with BOLD activation, suggesting regional synaptic activity may have diffuse effects on associated functional networks (Wey et al., 2014; McCutcheon et al., 2019). The degree to which RSN functioning may reflect systems of similar local neurophysiological composition or be influenced by systems of modulatory circuitry remains a critical question in understanding the neurophysiology of RSN activity.

The aim of the current study is to investigate associations between RSN functioning and a cell-level feature of brain physiology—synaptic density. Synaptic density can be reliably measured through the examination of the synaptic vesicle protein 2A (SV2A), the most monodispersed synaptic vesicle protein (Bajjalieh et al., 1994; Janz and Südhof, 1999; Mutch et al., 2011). PET imaging with the SV2A radioligand ^11^C-UCB-J has been validated as an indirect measure of synaptic density and is sensitive to neuron-level alterations in multiple psychiatric and neurodegenerative diseases (Finnema et al., 2016; Chen et al., 2018; Holmes et al., 2019; Matuskey et al., 2020; Mecca et al., 2020; D’Souza et al., 2021; Angarita et al., 2022). Synaptic density as assessed with ^11^C-UCB-J has been linked to measures reflecting local synaptic activity, such as glucose metabolism (Chen et al., 2021; van Aalst et al., 2021) that have themselves been associated with BOLD signaling (Tomasi et al., 2013). Potential links between synaptic density and RSN function have also been indicated by associations between synaptic biomarkers in CSF and functional connectivity changes in the default-mode network (Pereira et al., 2021). However, we have previously demonstrated through independent component analysis (ICA) of ^11^C-UCB-J PET data, that the sources of coherent spatial variance of synaptic density have limited spatial overlap with canonical RSNs (Fang et al., 2021), indicating that the organization of synaptic density variability differs, at least in part, from the functional organization of RSNs.

Here, cross-sectional analyses were performed to investigate potential within-subject associations between synaptic density networks and *a priori* selected canonical RSNs: the default-mode network, right frontoparietal executive-control, salience, and sensorimotor networks. These RSNs were selected because of their *spatial* and *functional* interrelationships, providing the opportunity to examine different potential associations with synaptic density networks. That is, these RSNs include functionally distinct networks with regional overlap (e.g., default-made and salience networks), spatially distinct networks with functional overlap (e.g., salience and executive-control networks), or spatially and functionally distinct networks (i.e., sensorimotor network). Similarly, conservative selection criteria were used to identify a limited set of ^11^C-UCB-J sources that encompassed regions with anatomical and functional associations to the RSNs of interest. We anticipated links between cellular and functional systems, such that greater intensity of ^11^C-UCB-J sources [indicating higher synaptic density, and thus greater potential for higher neural activity (Chen et al., 2021)], would be associated with higher fractional amplitudes of RSN activity. The fractional amplitude of low-frequency fluctuations (fALFF) is a common measure of RSN signal magnitude that is sensitive to alterations in resting-state brain activity in health and disease (Zou et al., 2008; Huang et al., 2017; Sato et al., 2019; Zhou et al., 2019; Vieira et al., 2020). Investigating all pairwise associations between the selected synaptic density networks and RSNs, we hypothesized strong links between pairs of spatially overlapping synaptic density networks and RSNs, and moderate links between spatially distinct but anatomically connected or functionally associated pairs. Finally, given prior findings of a shared negative association with age (Varangis et al., 2019; Fang et al., 2021), we explored the degree to which weakening synaptic density sources may mediate relationships between aging and declines in RSN activity.

## 2 Materials and methods

### 2.1 Participants

Data from 34 healthy adults (18M/16F, mean age: 46.4 ± 15.3 years) were included in this study. The sample was comprised of individuals included in a prior report of ^11^C-UCB-J binding that had also completed resting-state fMRI (Fang et al., 2021). Participants were screened using clinical interviews, physical examination with medical history, routine blood tests, electrocardiogram, and urine toxicology. PET and fMRI scans were completed 10.2 ± 30.4 days apart (with 28 subjects completing scans within 2 weeks). Participants provided written informed consent and did not meet the criteria for a current and/or lifetime psychiatric disorder, current or past serious medical or neurological illness, or have a history of substance abuse or dependence. Data were collected as part of multiple protocols approved by the Yale University Human Investigation Committee and participants provided informed consent.

### 2.2 MRI, acquisition, processing, and component analysis

MRI data were collected on a 3T MAGNETOM Prismafit scanner (Siemens, Erlangen, Germany) using two accelerated echo-planar imaging (EPI) sequences: multiband factor = 4, TR/TE = 1,000/30 ms, flip angle = 62°, resolution = 2 × 2 × 2 mm^3^, 60 slices, 300 s (*N* = 19) and multiband factor = 6, TR/TE = 1,000/30 ms, flip angle = 60°, resolution = 2 × 2 × 2 mm^3^, 72 slices, 282 s (*N* = 14). The first 282 s of eyes-open resting-state data were included for all participants. A standard high-resolution T1-weighted structural MPRAGE scan (TR/ TE = 2,530/3.34, flip angle = 7°, in-plane resolution = 0.98 × 0.98 mm, matrix size = 256 × 256, slice thickness = 1 mm, slices = 176) was also acquired during the same MR scanning session for image registration.

Spatial processing of fMRI data was performed using SPM12 (Wellcome Trust Centre for Neuroimaging, London, UK). Non-linear transformations from native subject space to MNI152 template space were determined from the high-resolution structural images using CAT12 for SPM (version 12.7^1^). Resting-state fMRI data were motion-corrected (mean framewise displacement was less than 0.5 for all subjects) and linearly registered to the subject’s structural image using default SPM12 settings prior to applying the non-linear transformation and spatial smoothing with an 8 mm FWHM Gaussian kernel.

ICA was performed on resting-state fMRI using the GroupICA Toolbox (GroupICAT v4.0c^2^). For ICA of the fMRI time series, data from the first 282 s of resting-state data for all participants were concatenated into a single group and reduced through principal component analysis prior to the extraction of 30 components using the InfoMax algorithm (Bell and Sejnowski, 1995) to identify established large-scale RSNs (Abou-Elseoud et al., 2010). The ICA was masked using the whole-brain template provided with CAT12, iterated 20 times using ICASSO to assess the stability and consistency of extracted components (Himberg et al., 2004). Group average spatial source maps were Z-scaled and component time courses were reconstructed using GICA3 to percent BOLD signal units for each subject (Erhardt et al., 2011). The *a priori* RSNs of interest were identified as the components with the maximum beta coefficient resulting from spatial regressions in relation to five RSN templates representing the anterior and posterior default mode, right frontoparietal executive-control network, the cinguloinsular salience network and the bilateral sensorimotor network (Allen et al., 2011) using the GIFT spatial sorting utility. Individual time courses were transferred to the frequency domain using multi-taper spectral estimation in GIFT and the fALFF for each network was calculated as the ratio of the integral of spectral power of low (0.01–0.08 Hz) to all (0.01–0.5 Hz) frequencies for each subject.

### 2.3 PET acquisition, processing, and component analysis

^11^C-UCB-J was synthesized as described previously (Nabulsi et al., 2016) and administered as an intravenous bolus injection over one minute (600.4 ± 148.1 MBq; inJ. mass: 1.22 ± 0.67 μg). Dynamic PET scans (HRRT, Siemens/CTI, Knoxville, TN, USA) were acquired in list mode (207 slices, 1.2 mm slice separation, reconstructed image resolution ~3 mm). Blood sampling was performed during PET scans using arterial catheters (drawn every 10 s for the first 90 s, then at 1.75, 2, 2.25, 2.5, 2.75, 3, 4, 5, 6, 8, 10, 15, 20, 25, 30, 45, 60 min after ^11^C-UCB-J injection) for metabolite analysis and determination of the plasma free fraction of ^11^C-UCB-J as described previously (Finnema et al., 2018). A transmission scan was also obtained for attenuation correction. Dynamic PET data (6 × 0.5 min, 3 × 1 min, 2 × 2 min, and 10 × 5 min) were reconstructed using the MOLAR algorithm with corrections for attenuation, normalization, scatter, randoms, deadline, and motion (Carson et al., 2003). Event-by-event head motion correction was included in the reconstruction based on motion detection with a Polaris Vicra optical tracking system (NDI Systems, Waterloo, Canada) using reflectors mounted on a cap worn by the subject (Jin et al., 2013). Parametric volume of distribution (*V*_T_) images using 60 min of dynamic data were generated with a one-tissue compartment model using the metabolite-corrected arterial plasma curve (Finnema et al., 2018).

Spatial processing of PET data was performed using SPM12. Parametric *V*_T_ images were linearly registered to the subject’s structural MR image using an intermediary early sum image (0–10 min post-injection) prior to applying the same non-linear transformation from CAT12 that was used for fMRI processing. Consistent with the methods used previously to identify replicable ^11^C-UCB-J *V*_T_ sources, *V*_T_ images were then smoothed with a 12 mm FWHM Gaussian (Fang et al., 2021).

ICA was performed on ^11^C-UCB-J *V*_T_ images using the GIFT Source-Based Morphometry toolbox (Xu et al., 2009). Parametric *V*_T_ images from all participants were concatenated into a “subject series”, which was reduced using principal component analysis prior to extracting 18 components consistent with our prior ICA of ^11^C-UCB-J in healthy adults (Fang et al., 2021). The ICA was iterated 50 times using ICASSO and group-average component spatial maps were Z-scaled for visualization. Three source networks were identified as meeting criteria as being primary sources (i.e., those accounting for >2% of total variance) that encompassed cortical and striatal regions of interest. Subject loadings for each selected component (i.e., the ICA beta-weight representing the intensity of contribution of each ^11^C-UCB-J source to an individual’s total regional *V*_T_) were used in subsequent analyses.

### 2.4 Statistical analysis

Primary hypotheses were tested using pairwise Pearson’s correlations between RSN fALFF and subject loadings on ^11^C-UCB-J networks using SPSS (v26.0, IBM Corp, Armonk, NY). Results for each ^11^C-UCB-J source network were examined using family-wise error correction at *p* < 0.05 (i.e., α/5 RSNs) and further explored at uncorrected *p* < 0.05. Similarly, pairwise correlations were performed to examine the relationship between RSN fALFF and ^11^C-UCB-J source loadings with age. Exploratory *post-hoc* mediation analyses were performed on the significantly intercorrelated relationships between age, ICA-estimated *V*_T_ in the medial prefrontal cortex and anterior default-mode network fALFF using PROCESS v4.0 (Hayes, 2017) for SPSS with 5,000 bootstrap resamples to handle the limited sample sizes. Models tested whether synaptic density was a potential mediator in the association between age and RSN activity and 95% confidence intervals that excluded zero determined significant mediations.

## 3 Results

**Figure 1:**
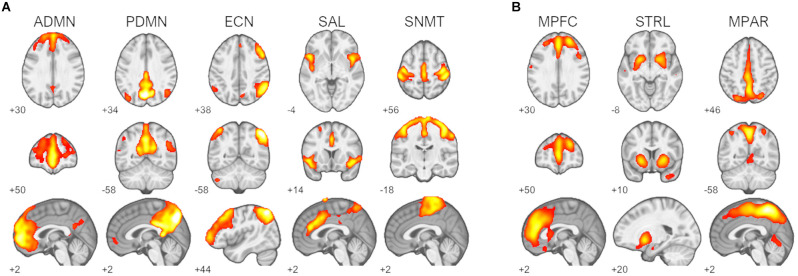
Identified resting-state networks (RSNs) and ^11^C-UCB-J sources. **(A)** RSNs of interest: default-mode networks (ADMN, anterior default-mode network; PDMN, posterior default-mode network), right frontoparietal executive-control network (ECN), salience network (SAL), and sensorimotor network (SNMT). **(B)** ^11^C-UCB-J sources of interest: medial prefrontal source (MPFC), striatal source (STRL), and medial parietal source MPAR. Z-scored spatial maps displayed at *z* > 2 (red) to *z* > 8 (white).

Inspection of component spatial sources derived from ICA of the resting-state fMRI data identified five functional brain networks representing *a priori* RSNs of interest (Figure 1A). Two components were consistent with the primary nodes of default-mode network, an anterior medial prefrontal RSN and a posterior parietal RSN. Single components were identified as being consistent with the right frontoparietal executive-control network, the cinguloinsular salience network, and the bilateral sensorimotor network. Three ^11^C-UCB-J *V*_T_ source networks of interest encompassing the medial prefrontal cortex, medial parietal cortex, and striatal regions were identified (Figure 1B).

**Figure 2:**
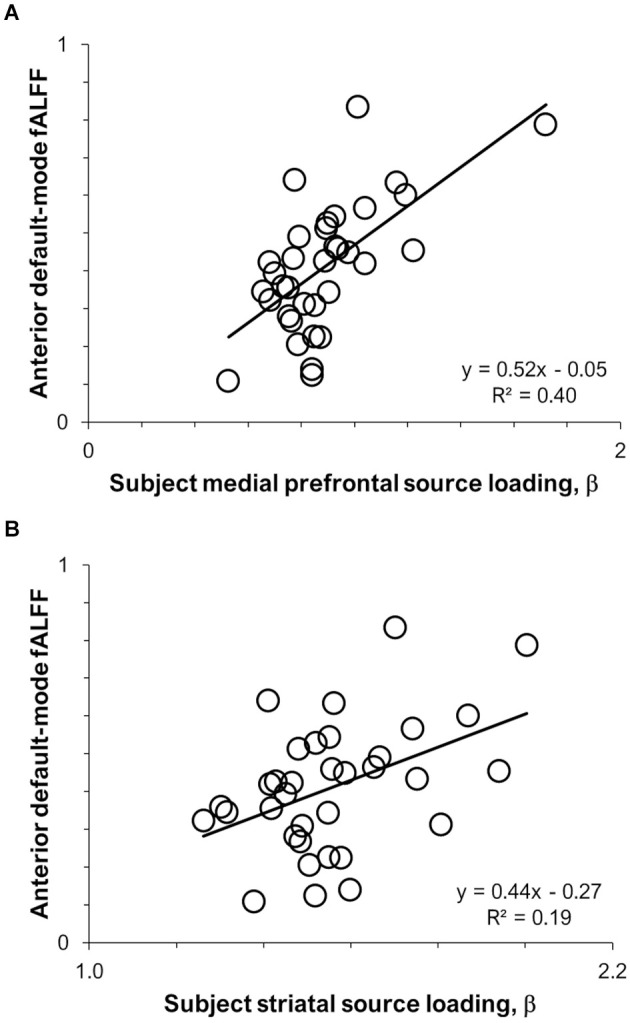
Scatterplots displaying intermodal relationships between **(A)** the medial prefrontal ^11^C-UCB-J source and anterior default-mode fALFF; and **(B)** the striatal ^11^C-UCB-J source and anterior default-mode fALFF.

Results of the pairwise correlations between ^11^C-UCB-J *V*_T_ sources and RSNs are provided in Table 1. The medial prefrontal ^11^C-UCB-J *V*_T_ source correlated positively with fALFF of the anterior default-mode network at the family-wise error corrected threshold (*r* = 0.63, *p* < 0.001; Figure 2A), and with fALFF of the posterior default-mode (*r* = 0.35, *p* = 0.040) and executive control (*r* = 0.36, *p* = 0.036) networks at uncorrected significance thresholds. The identified striatal ^11^C-UCB-J *V*_T_ source correlated positively with fALFF of the anterior default-mode network at the family-wise error corrected threshold (*r* = 0.44, *p* = 0.009; Figure 2B), and with fALFF of the salience network at uncorrected threshold (*r* = 0.34, *p* = 0.046). The medial parietal ^11^C-UCB-J *V*_T_ source was not correlated with fALFF values for any RSN of interest.

**Table 1:** Correlations (Pearson’s r) between ^11^C-UCB-J and RSN networks.

		^11^C-UCB-J source network	
Resting-state network	MPFC	STRL	MPAR
Anterior default-mode	0.634**	0.440**	−0.136
Posterior default-mode	0.354**	0.260**	−0.039
Executive control	0.361**	0.243**	−0.034
Salience	0.300**	0.344**	−0.031
Sensorimotor	0.230**	0.194**	−0.153

Abbreviations: MPFC, medial prefrontal cortex; STRL, striatal; MPAR, medial parietal. Underlined *r*-values indicate associations surviving family-wise error rate correction at *p* < 0.05. Uncorrected thresholds: *p < 0.05, **p < 0.01.

**Figure 3:**
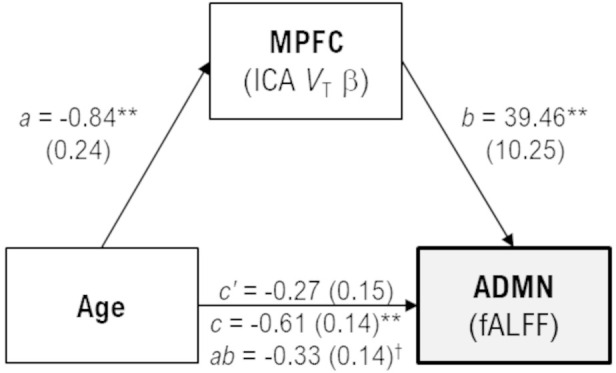
Mediation model with unstandardized coefficient values and standard error [B(SE)], scaled by 100 for display purposes. Age was indirectly related to anterior default-mode (ADMN) fALFF through its relationship with medial prefrontal (MPFC) *V*_T_. Greater age was associated with less MPFC *V*_T_ (*a, p* = 0.0014), and less MPFC *V*_T_ was associated with less ADMN fALFF (*b, p* < 0.001). While the direct effect of age on ADMN fALFF was not significant in the mediation model (*c’*, *p* = 0.064), there was a significant indirect effect of greater age on lower ADMN fALFF through MPFC *V*_T_ (*ab*, 95%CI = −0.62, −0.04) that accounted for approximately 54% of the total effect of age on ADMN fALFF (*c*, *p* < 0.001). ^†^95% confidence intervals do not include zero, indicating a significant effect. **p < 0.001.

In exploratory analyses, age was negatively correlated with fALFF values only for the anterior default-mode (*r* = −0.54, *p* < 0.001) and executive-control (*r* = −0.37, *p* = 0.030) networks, as well as subject loadings of only the medial prefrontal ^11^C-UCB-J source (*r* = −0.61, *p* < 0.001). *Post-hoc* analyses were performed to explore the extent to which ^11^C-UCB-J *V*_T_ (i.e., declines in synaptic density) may mediate the association between aging and decreasing activity in the anterior default-mode network (Figure 3). Age was indirectly related to anterior default-mode network fALFF through its relationship with medial prefrontal ^11^C-UCB-J *V*_T_ (unstandardized *B* = −0.0033, 95%CI = [−0.0062, −0.0004]), with the indirect effect accounting for approximately 54% of the total association between of age and anterior default-mode activity.

## 4 Discussion

To our knowledge, this study is the first multimodal study comparing ^11^C-UCB-J source networks and RSN activity in the same subjects. Our main aim was to investigate the degree to which RSN activity may reflect neuronal physiology, which is still partly unknown despite extensive research efforts. To do so, we examined RSN fALFF, a commonly used measure of the amplitude of spontaneous brain activity, as it relates to an *in vivo* measure of synaptic density, the distribution of synaptic protein SV2A from ^11^C-UCB-J PET (Rossi et al., 2022). Of note, fALFF has been found to be related to PET measures of glucose and oxygen metabolism, as well as cerebral blood flow (Aiello et al., 2015; Bernier et al., 2017; Deng et al., 2022). Using ICA to isolate both the activity of *a priori* RSNs and the intensity of ^11^C-UCB-J source networks, we provide evidence for a relationship between the brain’s functional organization and systems of synaptic density in healthy adults, for the first time.

In our *a priori* established functional and physiological systems of interest, RSN fALFF was distinctly associated with subject loadings of ^11^C-UCB-J sources both locally and along known anatomical and functional pathways. Specifically, greater medial prefrontal synaptic density was robustly associated with higher fALFF in the spatially overlapping anterior node of the default-mode network, but also the functionally associated posterior default-mode and right frontoparietal executive-control networks. Striatal synaptic density positively correlated with fALFF of the functionally associated default-mode and salience networks. Contrary to hypotheses, medial parietal synaptic density was not associated with fALFF of any RSN, and the sensorimotor RSN was not linked to any of the three ^11^C-UCB-J sources examined. These findings, although preliminary, expand our understanding of the possible physiological bases of RSN functioning.

Investigating the association between selected synaptic density and *spatially* related functional brain networks, we found a strong link between the medial prefrontal synaptic density network and the anterior default-mode network, which were nearly spatially identical. This is to some extent expected, given the known influences of local tissue properties, including gray matter and receptor density, on resting-state BOLD signal amplitudes (Wen et al., 2018; Garzón et al., 2021). By contrast, however, synaptic density in a parietal source network was not associated with the moderately spatially overlapping posterior node of the default-mode, or the partially overlapping sensorimotor network. In summary, evidence for a direct local association between synaptic density and RSN activity was mixed, suggesting the degree to which RSN amplitudes reflect underlying synaptic density systems may be regionally dependent in healthy adults. Notably, this regional heterogeneity in coupling is consistent with previous findings on associations between glucose metabolism and BOLD signals (Aiello et al., 2015; Wang et al., 2021).

Synaptic density systems were also related to spatially distant but *functionally* associated RSNs. Most robustly, greater striatal synaptic density was associated with higher fALFF of the anterior default mode network. In addition, the striatal source was associated with the salience network and the medial prefrontal source was associated with the right frontoparietal executive-control network. These RSNs form a three-network system implicated as the core functional system regulating internal cognitive processing and external sensory processing (Menon, 2011; Chand et al., 2017). The association between greater synaptic density in the striatum and the salience network is consistent with their role in switching the balance of the three-network system (Lehéricy et al., 2004a, b; Leh et al., 2007; Choi et al., 2012; Marek and Greene, 2021). On the basis of this, we can even speculate on possible functional distinctions between striatal synaptic density links to the default-mode and salience network mechanisms, on the one hand, and medial prefrontal synaptic density links to the opposing default-mode and executive-control mechanisms, on the other. Together, these findings reflect potentially *circuit-based* mechanisms in the relationship between local synaptic density and the functional activity of distant but functionally interconnected brain networks. On this, there is emerging literature suggesting that synaptic density and activity relate to large-scale RSN connectivity. A seminal study compared resting-state fMRI to spatially matched regional gene expression, and found that genetic variation (i.e., gene expression levels and common polymorphisms) was significantly related to RSN connectivity strength, with associated genes mainly responsible for ion channels (in particular, sodium channels), which are implicated in diseases such as Alzheimer’s and schizophrenia, and are tightly linked to synaptic function (Richiardi et al., 2015). These findings were later replicated (Vértes et al., 2016; Zhang et al., 2021), and point to a link between synaptic density and the modulation of RSN functional connectivity. Other relevant studies are those that relate RSN connectivity and measures of synaptic activity such as glucose metabolism (Aiello et al., 2015; Bernier et al., 2017; Deng et al., 2022; Palombit et al., 2022) or synaptic plasticity such as protein turnover (Hellyer et al., 2017). Overall, the pattern of associations between *functionally* associated pairs of ^11^C-UCB-J sources and RSNs is consistent with the presence of a modulatory, circuit-based influence of neuronal molecular activity on RSN functioning.

Contrary to the hypotheses, there were no links observed for either the medial parietal 11C-UCB-J source with any RSN, or the sensorimotor RSN with any ^11^C-UCB-J source. The lack of intermodal relationships of these two networks suggests associations between synaptic density and RSN activity may be regionally or functionally dependent. That is, while the medial parietal synaptic density source partially overlapped with the posterior default-mode and sensorimotor networks, the activity of these networks may be more heavily influenced by circuit-based mechanisms than by local synaptic density. Furthermore, despite well-established links between sensorimotor regions and cortico-subcortical circuitry (Lehéricy et al., 2004a, b; Leh et al., 2007; Alves et al., 2019), relationships between the activity of the sensorimotor RSN with this synaptic architecture may not be captured in the resting state, which perhaps offers a limited view of both sensory and motor functions. Future research examining associations between synaptic density sources and functional brain networks during task performance may provide further insight into potential intermodal relationships.

Exploratory analyses identified an interrelationship between the influence of aging on decreasing synaptic density and lower RSN activity (Varangis et al., 2019; Fang et al., 2021). Mediation analyses revealed the anticipated finding that weakening medial prefrontal synaptic density mediated the relationship between aging and a decline in default-mode activity. That is, while healthy aging is associated with a decline in default-mode network activity, age-related medial prefrontal synaptic density loss influences the degree to which aging affects default-mode blunting. This mediation effect of prefrontal synaptic density accounted for half of the association between aging and default-mode activity. Together these findings further support direct links between local neural physiology and RSN function, particularly within the medial prefrontal cortex.

There are limitations to this study that need to be considered. Relative to modern fMRI studies, the sample is small (*n* = 34), though it represents a substantial sample relative to typical PET studies. Moreover, a restricted set of canonical RSNs and ^11^C-UCB-J sources was selected *a priori* to test a limited set of specific hypotheses in this initial investigation. While this approach minimized the number of spurious correlations (Type I error), potential intermodal correlations between additional RSNs and ^11^C-UCB-J sources were not investigated. These limitations are best addressed in future studies with larger, potentially multi-site samples. Beyond replicating current findings in larger samples of healthy adults, the observed associations between synaptic density systems and RSN activity should be examined for relevance to disease pathologies such as Alzheimer’s or Parkinson’s diseases. In addition, fALFF was chosen as the resting-state fMRI measure of interest because we anticipated synaptic density would likely be related to signal amplitude; however, it may influence other aspects of resting-state activity, such as its local coherence, or large-scale functional connectivity, as previously tested with ^18^F-FDG metabolism (Aiello et al., 2015; Bernier et al., 2017; Deng et al., 2022). Similarly, the use of ICA to identify RSNs represents one of the multiple approaches to identify or characterize functional brain networks; for example, others have reported links between ^11^C-UCB-J binding and regional intrinsic connectivity density (Holmes et al., 2019). In this study, we used *V*_T_ as our PET outcome measure, as this is the primary outcome from kinetic modeling that is directly related to the synaptic density (Finnema et al., 2016, 2018). However, other outcome measures have been used. When appropriate, binding potential (*BP*_ND_) can be determined with an appropriate reference region either by scaling *V*_T_ values or by using reference modeling methods (Matuskey et al., 2020; Mecca et al., 2020). Alternatively, a simple tissue ratio, standardized uptake value ratio (SUVR), can provide accurate estimates of *BP*_ND_ without arterial sampling or dynamic scans (Naganawa et al., 2021). In future work, we will assess whether these other PET outcome measures provide comparable utility for comparison with fMRI metrics. Finally, we have previously demonstrated links between the ^11^C-UCB-J tracer delivery (i.e., *K*_1_) and task-based local BOLD amplitudes (Smart et al., 2021) that were not found with tracer binding (*V*_T_). Along with the previously mentioned glucose metabolism and protein turnover, these factors need to be kept in mind for understanding the complex relationships between neuronal synaptic physiology and functional brain activity (Yu et al., 2022).

Identifying links between RSN functioning and neural physiology at the molecular level is essential given the current ubiquity of resting-state fMRI research, and has particular importance for neurodegenerative and psychiatric disorders where both RSNs and molecular markers are found to be altered (He et al., 2007; Mecca et al., 2020). In this study, systems of coherent synaptic density in the medial prefrontal cortex and striatum were associated with greater RSN functional amplitudes in spatially overlapping cortical networks and in other cortical networks implying circuit-based relationships. These initial findings provide insight into potential links between large-scale synaptic architecture and intrinsic functional brain networks.

## Data availability statement

The raw data supporting the conclusions of this article will be made available by the authors, without undue reservation.

## Ethics statement

The studies involving human participants were reviewed and approved by Yale University Human Investigations Committee. The patients/participants provided their written informed consent to participate in this study.

## Author contributions

All authors contributed to the acquisition, analysis, and/or interpretation of data, drafting the work or revising it critically for intellectual content, and all provided final approval of the version to be published.

## Funding

This research was funded by the National Institutes of Health (R01 NS094253, R01 AG052560, K01 DA042998), Veterans Affairs National Center for PTSD, and the Nancy Taylor Foundation. Its contents are solely the responsibility of the authors and do not necessarily represent the official view of the funding agencies.

## Conflict of Interest

The authors declare that the research was conducted in the absence of any commercial or financial relationships that could be construed as a potential conflict of interest.

## Publisher’s Note

All claims expressed in this article are solely those of the authors and do not necessarily represent those of their affiliated organizations, or those of the publisher, the editors and the reviewers. Any product that may be evaluated in this article, or claim that may be made by its manufacturer, is not guaranteed or endorsed by the publisher.

## Footnotes

^1^ http://www.neuro.uni-jena.de/cat12/

^2^ https://trendscenter.org/software/gift/
